# Died

**Published:** 1883-01

**Authors:** 


Died.—On the morning of January 1st, at 5 o'clock, in Lancaster, Pa., Dr. Marshall H. Webb.

This sad event, though not a great surprise to those who knew of the state of the Doctor's health for some time past, will bring sorrow wherever he was known.

His friends were many; and wherever dentistry is known and practiced his name is familiar. He was intensely devoted to his profession, perhaps too much so, for his health and strength. He seemed to have little thought for himself, so long as he could summon strength sufficient to respond to the professional calls made upon him.

He put his whole energy into the development of one branch of his profession, and in this he was pre-eminently successful, but it cost him his life, and the profession the loss of one whom it could ill afford to spare.

His family will have the sincere sympathy of a host of friends. A more extended account of his life will hereafter be made.

